# Supplementary material for: Patterns of Intron Gain and Loss in Fungi
Source: PLoS Biol. 2004 Nov 30;2(12):e422. doi: 10.1371/journal.pbio.0020422 (PMC532390; doi:10.1371/journal.pbio.0020422)
Supplement: Table S1 — Also available at http://genes.mit.edu/NielsenEtAl/. (4.3 MB ZIP). [file pbio.0020422.st001.zip › NielsenEtAl/html/309.html]

AN4441.1.NCU03335.1.MG03907.1.FG01620.1


```
 CLUSTAL W (1.82) Multiple Sequence Alignments - Introns Inserted


Sequence 1: NCU03335.1	359 aa
Sequence 2: FG01620.1	360 aa
Sequence 3: MG03907.1	364 aa
Sequence 4: AN4441.1	339 aa
Alignment Length: 384 aa
Number Identitical Residues: 130 aa
Alignment Score (without introns) 7068


MG03907.1 	MSSAKATGANTSNSSSSGARSRDHNQGNQGRSFTPEQKEAVLRIRRCKPTAFYDILA--L
NCU03335.1	MPAATTSGADASN--GSSAHNRQHNQGNQERKYTAEQKAAVLRIRKCEPTAFYEIL---E
FG01620.1 	MPSATASGADT----GGSARSRDHKQGNQGRTFTPDQEAAVIRIRKCEPTAFYDVLNLET
AN4441.1  	MPSATSSGTDS----GGSGKSRDHNQGNQDRKYTPEQKAAVIRIRKCSATAFYEILA---
          	*.:*.::*:::    ....:.*:*:**** *.:*.:*: **:***:*..****::*    

MG03907.1 	TTKTCTDAEIKRAYRKQSLLTHPDKNGYEGADEAFKM~VSRAFSVLGDKEKREKFDRFGT
NCU03335.1	VSKTCTDAEIKKAYRKQSLLTHPDKNGHEHADEAFKM~VSRAFSVLGDKEKRDKYDRFGT
FG01620.1 	VKTTCTESEIKKAYRKQSLLTHPDKNGHPHADEAFKM~VSRAFGILGDKEKREKFDKFGG
AN4441.1  	VEKTATDSEIKKAYRKLSLVTHPDKNGYEGADEAFKM1VSRAFQVLSDSEKRARYDKFGG
          	. .*.*::***:**** **:*******:  ******* ***** :*.*.*** ::*:** 

MG03907.1 	DPDSRFESARAQNPFSGFAQRNAGGGG-GGGGGGGGFPGFAGGFEEEISPEEMFRRFFGG
NCU03335.1	DPDSRFASAQQQNPFAGFANRGAARAH-QGG-MWGDDA--------DISPEEMFARFFGG
FG01620.1 	DPDSRFASAQANNPF--FNQRAGGGMG-RGGPMFHD----------DLTPEEMFARFFGG
AN4441.1  	DPDSRFQPSSGPAGASPFGGGFSGGGFPRGGGGFEA----------EISPEELFNRFFNG
          	****** .:      : *    ..   . **               :::***:* ***.*

MG03907.1 	~G--GFGQ----FG-G1FDTGPQFVFNMGGGPGIRVHQFGGNRPRRRPRAEG---EPDPD
NCU03335.1	~G--GFGG--GPFGAA1FDGGPQFVFNFGGGPGIRVHQFGGGRPRTRPRQAG--QQQEGG
FG01620.1 	~GGGGFGGGGGPFG-A1FDTGPQFVFNFGGGPGIRVHQFGGGRPRTRPREAAGGARQEEG
AN4441.1  	1G--------------~----PQFVFNMGGGPGFRVHQFGGTQPRRRPRADA---QPEPT
          	 *                   ******:*****:******* :** ***  .   . :  

MG03907.1 	AGILSTLLGLLPILFLFIIPLISSIMSG----GSSSMPDVVFDEPQAPFTHKVKIPEHNV
NCU03335.1	NSMWSTLMSLLPIIILFIFPMISSLFSG--ATTGPSTPAMAFEQAIPPYTMARKMPNVKV
FG01620.1 	N-AFQTLLGLLPIILFFILPIITSFFSGDAGTSAASNPRMVYDNPLHPYTQERKMPNLNT
AN4441.1  	PSAASVFRQLLPLILLFLLPLLSSLFSS---GPAPSGPSYRFD-PIPPHTQQRTTPEIKI
          	 .  ..:  ***::::*::*:::*::*.     ..* *   :: .  *.*   . *: : 

MG03907.1 	HYFVRPTDWQQFKGNNHKIAQLNKHAHVTLVNTLTHRCNVERHREQQLREQAQGWFFQDP
NCU03335.1	NYYVNPNDVRHYSAS--KLYNLDQRAEHIFVQQLQQQCEREMTEKRRLIEEAQGWFRTDQ
FG01620.1 	PYYVNPDEVAKYSQN--KLNKLDRTAEHQLLRHLKSECENELMYQRRLEDAATGWFYQDP
AN4441.1  	DYFLNPKDVSDFSSR--EFRKLDKQVEVDYVKKLRYECEGEILARDRMIQDAQGWFFPDV
          	 *::.* :  .:.    :: :*:: ..   :. *  .*: *   . :: : * ***  * 

MG03907.1 	EKMKIADQFKKPACERLSKL---ATRF
NCU03335.1	EKLAKAERFETPACIRIKNLYSPGSKY
FG01620.1 	DKMALAQSYSKPSCERLRNL---GVKF
AN4441.1  	EKIKEAKAMELKSCRRLESLP-LKRKY
          	:*:  *.  .  :* *: .*     ::
```
